# The temporal and stimuli-specific effects of LPS and IFNγ on microglial activation

**DOI:** 10.3389/fnagi.2026.1756410

**Published:** 2026-01-29

**Authors:** Christina N. Heiss, Andrew S. Naylor, Ida Pesämaa, Arketa Meshi, Benjamin Céspedes-Cortés, Chiméne Lounès, Ayat Taki, Katarina Türner Stenström, Dzeneta Vizlin-Hodzic, Henrik Zetterberg, Verónica Palma, Gunnar Brinkmalm, Ann Brinkmalm, Stefanie Fruhwürth

**Affiliations:** 1 Department of Psychiatry and Neurochemistry, Institute of Neuroscience and Physiology, The Sahlgrenska Academy, University of Gothenburg, Gothenburg, Sweden; 2 Laboratory of Stem Cells and Developmental Biology, Faculty of Science, University of Chile Santiago, Chile; 3 Clinical Neurochemistry Laboratory, Sahlgrenska University Hospital, Mölndal, Sweden; 4 Department of Neurodegenerative Disease, UCL Institute of Neurology, London, United Kingdom; 5 UK Dementia Research Institute at UCL, London, United Kingdom; 6 Department of Pathology and Laboratory Medicine, University of Wisconsin School of Medicine and Public Health, Madison, WI, United States; 7 Wisconsin Alzheimer's Disease Research Center, University of Wisconsin School of Medicine and Public Health, University of Wisconsin-Madison, Madison, WI, United States; 8 Centre for Brain Research, Indian Institute of Science, Bangalore, India

**Keywords:** DAM signature, interferon γ, LPS, microglia, neuroinflammation

## Abstract

Microglia, the resident immune cells of the central nervous system (CNS), play a pivotal role in health and disease maintaining homeostasis and mediating neuroinflammatory responses. Their activation is a dynamic and context-dependent process characterized by diverse phenotypic states defined by transcriptomic, proteomic, and morphological characteristics. While lipopolysaccharide (LPS) is widely used as an inflammatory stimulus in microglial research, its physiological relevance remains debated. Interferon gamma (IFNγ), a key pro-inflammatory cytokine involved in immune priming, more closely mimics CNS inflammatory conditions. In this study, we systematically investigated the temporal activation profiles of human iPSC-derived microglia (hiMG) in response to LPS, IFNγ, and their combination. Transcriptomic analysis at 24 h revealed robust differential gene expression, with over 7,000 genes altered by LPS and more than 8,500 by LPS/IFNγ co-stimulation. These profiles partially overlapped with disease-associated microglia (DAM) signatures, including upregulation of *S100A9*, *CD44*, *ACSL1*, and *HIF1A*, and downregulation of *TREM2*, *GPNMB*, *FABP3*, *LGMN*, and *LPL*. Cytokine expression changes were detectable as early as 1 h post-treatment, predominantly following LPS exposure, and displayed distinct early (≤2 h), mid (4–12 h), and late (24–96 h) temporal patterns. IFNγ alone induced modest transcriptomic and cytokine responses but contributed to sustained inflammatory signatures when combined with LPS. Morphological analysis showed marked LPS- and LPS/IFNγ-induced structural remodeling of hiMG consistent with activation. To assess protein-level dynamics, targeted mass spectrometry quantified secreted ApoE, CD44, FUCA1, Galectin-3, and Osteopontin, all relevant to microglial activation, which were compared to cellular protein expression measured by western blot. Time-dependent increases were most prominent following LPS and LPS/IFNγ treatment, although secreted Osteopontin levels were highest with IFNγ alone, highlighting stimulus-specific effects. Collectively, these data demonstrate that microglial activation is highly time- and stimulus-dependent, with LPS eliciting the strongest responses, and IFNγ modulating these effects. Our findings underscore the importance of temporal resolution in modeling microglial activation and provide insight into the mechanistic underpinnings of microglial activation relevant to neurodegeneration and therapeutic targeting.

## Introduction

The field of microglia research continues to flourish, with clear evidence that these brain-resident macrophages play a critical role in the onset and progression of neurodegenerative diseases (Gao et al., 2023). As a result, microglia-targeting therapies are emerging as strategies to modulate disease-associated neuroimmune responses (Gao et al., 2023; Mahmood and Miron, 2022; Noh et al., 2025). Microglial activation is a highly dynamic process, driven by their continuous surveillance of the local environment and characterized by phenotypic states that shift in response to environmental cues (Depp et al., 2025; Paolicelli et al., 2022; Snijders et al., 2025). These phenotypic states can be defined by multiple parameters including transcriptomic and proteomic signatures, metabolic adaptations, and morphological characteristics (Depp et al., 2025; Paolicelli et al., 2022). For example, during central nervous system (CNS) disease pathogenesis, microglia transition from homeostatic to reactive states, initially described as disease-associated microglia (DAMs) in a mouse model of Alzheimer’s disease (Keren-Shaul et al., 2017). Classical DAM signature genes are *TREM2*, *APOE*, *LPL*, *CD9*, *SPP1*, *FABP3*, *LGALS3*, and *GPNMB*. Another parameter is microglial morphology which is strongly influenced by context and signaling cues and can range from ramified, rod-like, hypertrophic, and ameboid forms. While ramified microglia were traditionally believed to be in a resting state and ameboid microglia were believed to be phagocytic, it is now clear that morphology does not equal function (Paolicelli et al., 2022).

Accumulating evidence demonstrates that prolonged microglial activation can shift from protective to detrimental, creating a toxic environment that damages otherwise healthy cells and tissues (Gao et al., 2023). A deeper understanding of microglial phenotypes, accounting for temporal and stimuli-dependent dynamics, enables more precise characterization of these protective and detrimental functions. These insights may enhance the precision and therapeutic potential of neuroimmune modulatory therapies.

Lipopolysaccharide (LPS) and interferon gamma (IFNγ) are commonly used to stimulate microglial activation, often with a priming purpose to pre-activate the cells prior to a long-lasting stimulus (Neher and Cunningham, 2019; Perry and Holmes, 2014; Stoberl et al., 2023). LPS is a potent trigger of innate immune activation, as these glycosylated lipids form a major component of the outer membrane of Gram-negative bacteria. The human immune system is highly sensitive to these endotoxin-classified molecules (Brinkworth and Valizadegan, 2021), which can induce sepsis and compromise blood–brain-barrier integrity (Aborode et al., 2025). However, the physiological relevance of LPS as a trigger of microglial activation and neuroinflammation remains uncertain and widely debated. To better mimic physiological conditions in the CNS, pro-inflammatory cytokines, particularly IFNγ, are commonly used to induce microglial activation (Kann et al., 2022; Stoberl et al., 2023). IFNγ is a type II interferon, known to promote inflammatory responses via several pathways, including metabolic reprogramming, nuclear factor kappa-B (NfkB)-mediated induction of proinflammatory factors such as tumor necrosis factor (Tnf) and interleukin-6 (Il-6), and increased production of nitric oxide (NO) (Ding et al., 1988; Ivashkiv, 2018; Riquelme et al., 2013; Wang et al., 2018). IFNγ priming has been shown to promote a sustained inflammatory response in microglia (Hemmerich et al., 2022; Papageorgiou et al., 2016; Perry and Holmes, 2014).

In this study, we characterize the effects of LPS and IFNγ, and their combined use, on the activation dynamics of human induced pluripotent stem cell (hiPSC)-derived microglia (hiMG) over a 96-h time course. At 24 h, transcriptomic analysis revealed more than 7,000 differentially expressed genes (DEGs) following LPS treatment, and over 8,500 DEGs following LPS/IFNγ treatment. Notably, the identified DEGs partially overlap with established human DAM signatures (Mancuso et al., 2024), including both upregulated and downregulated genes. Time-dependent increases in expression levels of different cytokines and chemokines were observed early (detectable changes within 2 h post treatment), mid (4–12 h post treatment), and late (24–96 h post treatment). Furthermore, targeted mass spectrometry was used to measure time-dependent secretion of five proteins which we defined to be relevant to microglial activation. These data were compared with cellular protein levels, assessed by western blot, to obtain a comprehensive understanding of the dynamics between cellular protein expression and release across time and treatment-specific conditions.

Collectively, our findings reveal that microglial activation unfolds in a distinctly time-dependent manner, shaped predominantly by LPS-driven signaling yet markedly modulated by IFNγ. These findings deepen our understanding of how inflammatory cues shape microglial states over time and emphasize the importance of considering temporal dynamics when studying microglial responses.

## Materials and methods

### Generation of human hiPSC-derived microglia (hiMG)

The human control iPSC lines WTSIi015-A (EBiSC through Sigma-Aldrich) and BIONi010-C (EBiSC through Sigma-Aldrich) were used throughout the experiments. Unless stated otherwise, the WTSli015-A line was used. hiMG were obtained as previously described with some modifications (Fruhwurth et al., 2023). hiPSCs were maintained on Matrigel (Corning) in mTeSR1 + medium (STEMCELL Technologies). hiPSC colonies were dissociated into single cells using TrypLE Express (Thermo Fisher Scientific). Per AggreWell 800 (STEMCELL Technologies) 24-well plate, 4 × 10^6^ hiPSCs were seeded in 2 mL of embryonic body medium (EBM). EBM consisted of mTeSR1 + medium supplemented with 10 μM ROCK inhibitor, BMP-4 (50 ng/mL), SCF (20 ng/mL), and VEGF_121_ (50 ng/mL) (all from PeproTech). Cells were cultured for 4 days in AggreWells to form embryonic bodies (EBs) with half-medium change (1 mL) every day. EBs were harvested using an inverted cell strainer (40 μm) and around 15 EBs were plated per six-well in hematopoietic medium (HM). HM consisted of X-VIVO 15 medium (Lonza) supplemented with 2 mM GlutaMAX, penicillin (100 U/mL), streptomycin (100 μg/mL), 55 μM β-mercaptoethanol, M-CSF (100 ng/mL) (PeproTech) and IL-3 (25 ng/mL) (PeproTech). Every 7 days, 2 mL of medium was replaced with fresh HM. After around 30 days, primitive macrophage precursors could be continuously harvested during the medium change and plated in microglia medium (MiM) at a density of 5 × 10^4^ cells/cm^2^. Precursors of good quality have a viability of 100% over the course of at least 2 months. Precursors were harvested for 2–3 months as long as the viability was 100% and the weekly cell count did not decrease. We did not observe significant differences in cell count or microglial activation profiles between differentiation batches. Biological replicates refer to separately treated cell culture wells of the same experiment. MiM consisted of Advanced DMEM F12 medium (Gibco) supplemented with 2 mM GlutaMAX, penicillin (100 U/mL), streptomycin (100 μg/mL), 55 μM β-mercaptoethanol, IL-34 (100 ng/mL) (PeproTech), and GM-CSF (10 ng/mL) (PeproTech). Finally, cells were differentiated in MiM for 6–9 days with full medium change every other day. For LDH cytotoxicity assay and targeted mass spectrometry experiments, phenol red-free MiM was used, consisting of phenol red-free DMEM medium (Gibco) supplemented with 1 mM Sodium Pyruvate (Sigma Aldrich), penicillin (100 U/mL), streptomycin (100 μg/mL), 55 μM β-mercaptoethanol, IL-34 (100 ng/mL), and GM-CSF (10 ng/mL).

### Generation of human hiPSC-derived neurons

hiPSC-derived neurons were obtained as previously described with some modifications (Fruhwurth et al., 2023). WTSIi015-A hiPSCs were passaged using EDTA (Thermo Fisher Scientific) and pooled 2:1 upon 100% confluency. The next day, medium was switched to neural induction medium (NIM) which consisted of neural maintenance medium (NMM) supplemented with mouse Noggin/CF chimera (500 ng/mL) (R&D Systems), and 10 μM SB431542 (STEMCELL Technologies). NMM consisted of DMEM/F12 and neurobasal medium (1:1) supplemented with 1× N2 supplement, 1× B27 supplement, 50 μM β-mercaptoethanol, 0.5× non-essential amino acids, 100 μM l-glutamine (all from Life Technologies), penicillin (100 U/mL), streptomycin (100 μg/mL), insulin (10 μg/mL), 0.5 mM sodium pyruvate (both from Sigma-Aldrich). Cells were maintained in NIM for 10–12 days and were then dissociated in colonies using dispase II (10 mg/mL) (Thermo Fisher Scientific) and seeded on laminin-coated plates (1–2 μg/cm^2^; Sigma-Aldrich) in NMM supplemented with FGF2 (20 ng/mL) (PeproTech). Cells were kept in FGF2-supplemented NMM for 4–5 days and then further passaged with dispase upon confluency until neurogenesis can be observed between day 20–25. The colonies were thereafter passaged and expanded using StemPro Accutase (Thermo Fisher Scientific) until day 35. On day 35, cells were passaged a final time and seeded on Ibidi μ-slides (Ibidi) coated with laminin (1–2 μg/cm^2^) at a density of 5 × 10^4^ cells/cm^2^ in NMM. Around day 49, hiMG precursors were added, 4 × 10^4^ cells/cm^2^, to establish co-cultures with evenly spaced microglia. Co-cultures were maintained in NMM supplemented with IL-34 and GM-CSF and used for experiments around day 63.

### Pro-inflammatory stimulation of hiMG and co-cultures

hiMG and co-cultures were incubated with LPS from *Escherichia coli* 026:B6 (eBioscience) at 100 ng/mL or IFNγ (preprotech) at 20 ng/mL or in combination LPS/IFNγ (LPS 100 ng/mL and IFNγ 20 ng/mL) and harvested at the indicated time points.

### RNA isolation, RT-PCR, and qPCR

hiMG were grown in 48-well plates and lysed in RLT Plus buffer (Qiagen) supplemented with 40 mM dithiothreitol (DTT) (Sigma) and RNA was isolated using the RNeasy Mini Kit (Qiagen) according to the manufacturer’s instructions. cDNA was synthesized using the High-Capacity cDNA Kit (Thermo Fisher Scientific). Quantitative polymerase chain reaction (qPCR) was performed using the following TaqMan Gene Expression Assays (Applied Biosystems): ACTB (Hs01060665_g1), IL1B (Hs01555410_m1), IL6 (Hs00174131_m1), TNFA (Hs00174128_m1), CXCL8 (Hs00174103_m1), CXCL10 (Hs00171042_m1), CCL2 (Hs00234140_m1). mRNA levels of interest were normalized to the housekeeping gene *ACTB*, and fold changes were calculated using the ΔΔCt method. To compare temporal changes within each treatment group, a timepoint 0 group was used as reference group. To compare groups at timepoints of interest, a Control (Ctrl) group was used as reference group for each respective timepoint.

### RNA sequencing and data analysis

hiMG were grown on 24-well plates and treated with LPS or LPS/IFNγ in MiM for 24 h. RNA was extracted using the RNeasy Mini Kit (Qiagen) with on-column DNase digestion (Qiagen). Around 500 ng of purified RNA were sent for bulk RNA sequencing (25 M reads per sample) to the Clinical Genomics unit (SciLifeLab Gothenburg). Analysis of FASTQ files was performed in R Studio version 4.4.1. Differential expression analysis and normalization were done using the DESeq2 package. The packages biomaRt, AnnotationDbi, and org.Hs.eg.db were used for gene annotation. Data visualization was done using the packages pheatmap, ggplot2, RColorBrewer, EnhancedVolcano, ggpubr, and ggrepel while data wrangling utilized the packages stringr, dplyr, and readxl. Raw read counts and sample info were imported into R. A DESeq-dataset with design ~condition was constructed. Variance-stabilized counts were obtained and used for quality control. Separation by condition was visualized with a principal component analysis (PCA) plot and Euclidean sample-to-sample distance was visualized with a sample distance heatmap. Differential expression analysis was performed using DESeq, which fits generalized linear models based on the negative binomial distribution. Wald tests were applied to assess significance of log2 fold changes between groups. Pairwise contrasts were created for LPS/IFNγ vs. Ctrl, LPS vs. Ctrl, and LPS/IFNγ vs. LPS. Genes with adjusted *p*-value (Benjamini-Hochberg) < 0.05 were considered significantly differentially expressed. Positive log2 fold changes indicate higher expression in the first group contrast (LPS/IFNγ or LPS) compared to the second (Ctrl or LPS). For functional interpretation, DESseq results of the pairwise comparisons were annotated with external_gene_name and description. The annotated data was then further analyzed and visualized as heatmaps, volcano plots, and Venn diagrams. Venn diagrams were generated for differentially expressed genes (log2Foldchange > 0 and <0). Heatmaps depict rlog-transformed expression values relative to the Ctrl group unless stated differently in the figure legend. Top genes were selected after adjusted p-value and log2 fold change criteria. A curated list of disease-associated microglia (DAM) genes was obtained from Mancuso et al. (2024). Genes present in at least three of the four referenced publications were included (403), of which 400 were identified in our annotated dataset.

### LDH cytotoxicity assay

hiMG were left untreated (Ctrl) or stimulated as described above (LPS, IFNγ, or LPS/IFNγ) for 24 h, 48 h, 72 h or 96 h in phenol-red-free MiM. The CyQUANT LDH cytotoxicity assay (Invitrogen) was used to measure cell cytotoxicity according to the manufacturer’s instructions. Briefly, after the different timepoints, 10 μL of lysis buffer was added to three wells to measure maximum LDH and 10 μL sterile water was added to all the other wells (spontaneous LDH controls and experimental wells). Cells were incubated for 45 min at 37 °C and then 50 μL cell supernatant from each well was transferred to a new plate together with 50 μL of reaction mixture and incubated at room temperature for 30 min protected from light. The reaction was terminated by the addition of stop solution, whereupon absorbance (A) was measured at 490 and 690 nm. Specific absorbance was calculated by subtracting background A (690 nm) from the A (490 nm) absorbance and the % cytotoxicity was calculated using the following formula:


$$\text{Cytotoxicity}\;[\%]=\frac{(\begin{array}{l} \text{experimental wells}\;\mathrm{LDH}\;\text{activity}-\\ \text{spontaneous}\;\mathrm{LDH}\;\text{activity} \end{array})\;}{\left(\begin{array}{l} \text{maximum}\;\mathrm{LDH}\;\text{activity}-\\ \text{spontaneous}\;\mathrm{LDH}\;\text{activity} \end{array}\right)}\times 100$$

### Immunocytochemistry

hiMG were grown on 48-well plates on 8 mm coverslips (Electron Microscopy Sciences), washed twice in PBS and fixed using Histofix (HistoLab) for 20 min at room temperature. Samples were washed twice in PBS, permeabilized with 0.3% Triton X-100 in tris-buffered saline (TBS) for 15 min at room temperature and incubated with blocking buffer (0.3% Triton X-100 and 5% donkey serum in TBS) for 1 h at room temperature. The primary antibodies IBA1 (1:500; 234017, SySy), TUJ1 (1:1,000; Abcam 18207), and SV2A (1:500; DSHB AB_2315387) were diluted in blocking buffer. Samples were incubated with primary antibodies overnight at 4 °C. Cells were washed three times in TBS and incubated for 1.5 h with secondary antibodies diluted 1:500 in blocking buffer. Cells were washed three times in TBS, counterstained using DAPI, and mounted using ProLong Gold mounting media. Samples were imaged using a Nikon A1 inverted confocal microscope. For cocultures, cells were grown on Ibidi μ-slides, stained as described above, and mounted with Ibidi mounting media (Micromedic).

### Morphology analysis

Co-cultures were treated with LPS/IFNγ for 24 h, followed by fixation and staining as described above. Samples were imaged using the 10× objective and 5 μm stepwise z-stacks. A total of three images per well were taken, with all samples blinded to ensure unbiased acquisition. The following morphology analysis pipeline was adapted from Kim et al. (2024). The different channels were separated using Fiji Image J and maximum projection was applied on the IBA1 single-channel images for further analysis. Prior to full processing, a representative image from the dataset was selected for parameter optimisation. Next, the BioVoxxel plugin was used to select the best thresholding option which allows for single-cell separation while still maintaining branching resolution, opting to reduce noise outside the threshold signal. The auto thresholding method Li was selected for this specific analysis. The MicrogliaMorphology Program plugin was utilized to isolate individual microglial cells and exclude background artifacts. The plugin segmented and skeletonized individual cells to compute branch lengths, branch points, and endpoints, as described in Kim et al. (2024). In total, 805 cells were detected and assessed using the MicrogliaMorphology Program and FracLac analysis for a total of 30 parameters, of which 28 were numeric variables and used for downstream analysis (Supplementary Figure 2D). The generated results were processed using the MicrogliamorphologyR pipeline (Kim et al., 2024) in R studio (R 4.5.1). The packages dplyr, tidyr, and tibble were used for data wrangling, while factoextra and ppclust were used for clustering and dimensionality-reduction. Moreover, the packages ggplot2, gridExtra, pheatmap, ggpubr, and scales were used for statistical modeling, whereas data was visualized using glmmTMB, DHARMa, Hmisc, car, multcomp, and emmeans. All parameters were merged into one data frame and PCA was performed on the logarithmically scaled data, generating 10 principal components. Of those, the first three explain around 80% of the data variability and were thus used to perform the k-means clustering, for morphological categorization of the clustered single cells as either ameboid, rod-like, hypertrophic, or ramified. To ensure correct definition of each cluster, the latter were correlated with the mean of each principal component (which represented specific morphological features) and directly correlated with all the features (Supplementary Figure 2E). Cluster composition was visualized using stacked bar plots. The differences in cluster abundance were assessed using a linear regression model (percentage [%] ≈ Condition × Cluster) fitted to image-level data. Model adequacy was evaluated using DHARMa’s simulated quantile residuals, where tests for uniformity, dispersion and outliers indicated no significant deviations. Moreover, individual morphological parameters (e.g., circularity) were compared between the control and LPS/IFNγ treated cells using two-sample *t*-tests and visualized using violin/boxplots.

### Targeted mass spectrometry (MS)

Using our newly developed liquid chromatography-mass spectrometry (LC–MS) method based on parallel reaction monitoring (PRM), five microglia-relevant proteins were reliably measured in the conditioned media from hiMG (Supplementary Table 1). hiMG were grown on 48-well plates, treated for the indicated time points, media was removed, immediately centrifuged at 400 *g* for 5 min, and supernatants were stored at −80 °C. In brief, samples were thawed at room temperature, vortexed, and briefly centrifuged before aliquoted (100 μL) into a Protein LoBind tube (Eppendorf). 25 μL of our established mix of custom stable-isotope-labeled peptides (SpikeTides, JPT) was added to each sample. DTT was diluted in 50 mM ammonium bicarbonate (ABC) to reach a final concentration of 30 mM. To reduce disulfide bonds, 25 μL of 30 mM DTT were added to each sample and incubated at 60 °C, 900 rpm, for 30 min. Following incubation, samples were cooled down to room temperature and briefly centrifuged. Indole-3-acetic acid (IAA) was diluted in 50 mM ABC to reach a final concentration of 70 mM. To alkylate cysteine residues, 25 μL of 70 mM IAA was added to each sample and incubated in the dark at room temperature, 600 rpm, for 30 min. Following incubation, proteolytic digestion was initiated by the addition of 25 μL Trypsin/Lys-C mix (Promega) and incubated at 37 °C, 900 rpm, overnight (approximately 18 h). Samples were briefly centrifuged and acidified by adding 25 μL 10% formic acid (FA) (Thermo Fisher Scientific). Following acidification, samples were vortexed and briefly centrifuged before desalting using solid phase extraction (SPE) using a 96-well Oasis HLB μElution 30 μm Plate (Waters). The yielded eluate, containing the digested peptides as well as the SpikeTide internal standard peptides, was collected in V-bottomed NC/AN Screw Cap tubes (Nordic Biolabs), dried using vacuum centrifugation, and stored at −80 °C. Prior to analysis using PRM-MS, samples were dissolved in 100 μL of 50 mM ABC, 600 rpm, for 20 min. For the proteomic analysis, the dissolved samples were loaded onto a Hypersil Gold Reverse Phase column (100 × 2.1 mm, 1.9 μm, Thermo Fisher Scientific), connected to a micro-LC MS Dionex 3000 system (300 μL/min, sample cycle time: 32 min, retention time window: 1 min) with electrospray ionization (ESI) coupled to a Q-Exactive mass spectrometer (Thermo Fisher Scientific). Mobile phases used: (A) FA (0.1%) and (B) acetonitrile solution (84%, containing FA (0.1%)), with a gradual increase of the acetonitrile solution for the elution of the peptides. The gradient went from 0 to 40% B over the time of 32 min. Mass spectra were acquired using a scheduled PRM method in accordance the following settings: retention time window per peptide: 1 min; isolation window: 3 *m/z*; automatic gain control target valve: 3 × 10^6^; maximum injection time 250 ms; resolution setting: 70,000.

### Immunoblotting

hiMG were grown on 12-well plates for protein isolation. After the treatment, cells were washed twice with cold PBS and lysed on ice for 5 min in radioimmunoprecipitation assay (RIPA) buffer [20 mM tris–HCl (pH 7.5), 150 mM NaCl, 1 mM EDTA, 1% Triton X-100, 0.5% sodium deoxycholate, 0.1% SDS] supplemented with protease and phosphatase inhibitor cocktails (Roche). Samples were sonicated on ice for 10 min and centrifuged at 14,000 *g* at 4 °C for 10 min. Supernatants were collected, boiled at 95 °C for 5 min under reducing conditions, and subjected to immunoblotting. The following primary antibodies were used: CD44 (CS37259, Cell Signaling Technology), FUCA1 (16420-1-AP, Proteintech), Galectin-3 (culture supernatant of the hybridoma M3/38 (Sundqvist et al., 2018); a generous gift from Assoc. Prof. Martina Sundqvist), OPN (CS66614, Cell Signaling Technology), and beta-Actin (CS3700, Cell Signaling Technology). Membranes were stripped using Restore PLUS western blot stripping buffer (Thermo Fisher Scientific) and reprobed. The following secondary antibodies were used: IRDye 800CW donkey anti-rabbit IgG, IRDye 680rd donkey anti-mouse IgG, and IRDye 800CW goat anti-rat IgG (all from LI-COR Biotechnology).

### Statistical analysis

Statistical analysis was performed using Graphpad Prism 10 software. Comparisons were carried out on multiple groups and timepoints using two-way analysis of variance (ANOVA) with multiple comparisons, followed by Tukey’s *post hoc* test with corrected *p*-values when all groups were compared against each other and by Dunnett’s post hoc test when comparisons were made against a single control group. Specific analyses are indicated in the figure legends. No measurement was excluded for statistical analysis.

## Results

### LPS and LPS/IFNγ drive overlapping as well as distinct transcriptional alterations in human microglia

**Figure 1:**
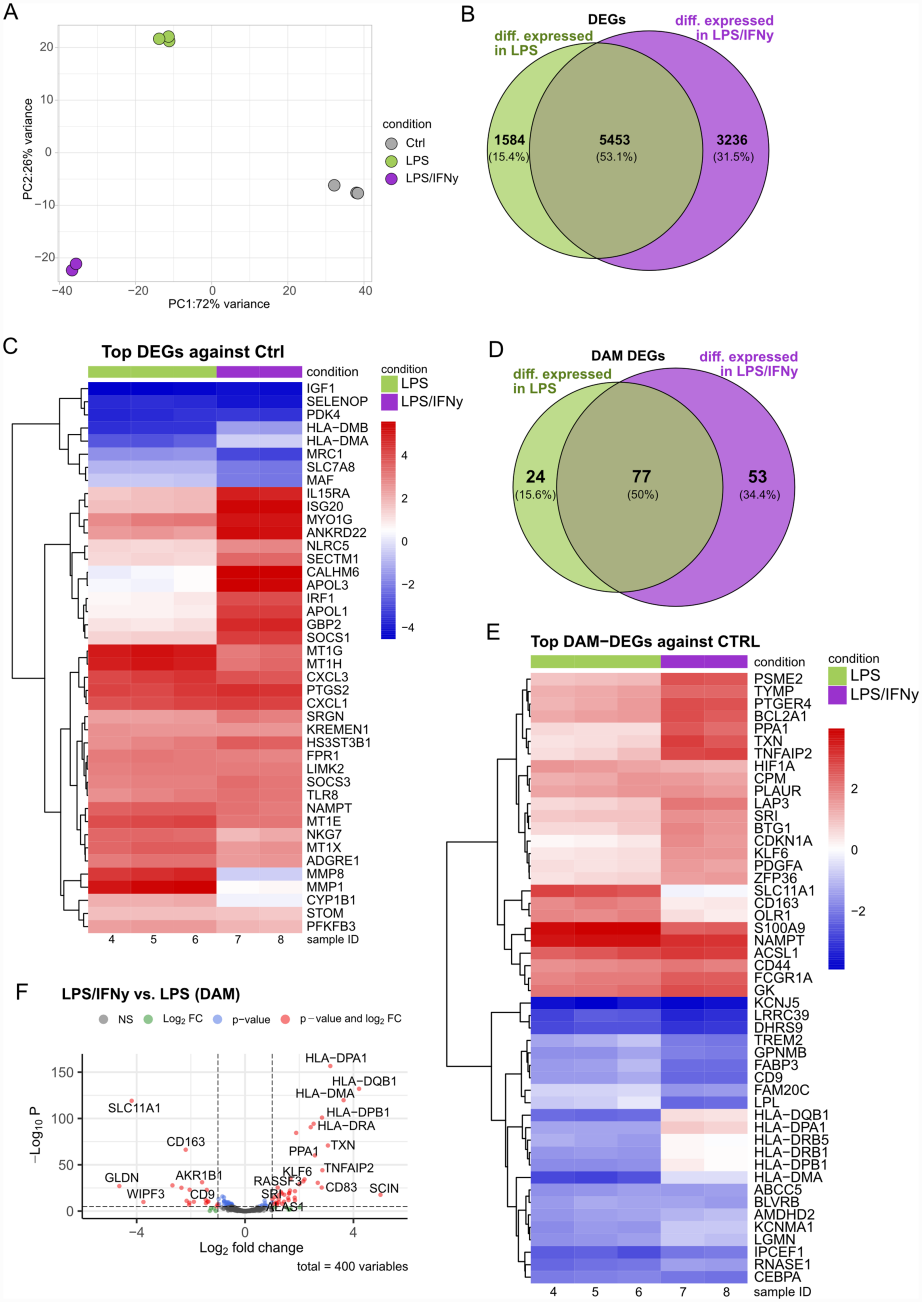
LPS and LPS/IFNγ treatments induce distinct gene expression signatures in human microglia. **(A)** PCA plot showing the separation between treatment groups and control group. **(B)** Venn diagram showing the overlap of significant DEGs between LPS vs. Ctrl and LPS/IFNγ vs. Ctrl. **(C)** Heatmap showing TOP DEGs in LPS and LPS/IFNγ. **(D)** Venn diagram showing the overlap of significantly differentially expressed DAM-genes between LPS/IFNγ vs. Ctrl and LPS vs. Ctrl. **(E)** Heatmap of the top DAM DEGs identified in the comparisons LPS vs. Ctrl and LPS/IFNγ vs. Ctrl. **(F)** Volcano plot of DAM-DEGs in the comparison LPS/IFNγ vs. LPS. *n* = 2–3 biological replicates of the same differentiation.

hiMG were stimulated for 24 h with either 100 ng/mL LPS alone or a combination of LPS and 20 ng/mL IFNγ, widely used concentrations (Stoberl et al., 2023), and compared to untreated controls. This experiment was designed specifically to assess the impact of IFNγ on the LPS-induced response and consequently did not include an IFNγ-only condition. We performed RNA sequencing to capture transcriptomic alterations between the groups. As revealed by PCA, the different treatment groups were clearly separated from each other, with LPS as a major driver of variance (Figure 1A). Samples within each group tightly clustered together (Figure 1A; Supplementary Figure 1A). LPS treatment induced >7,000 significant differential expression of genes (DEGs), while LPS/IFNγ results in >8,000 DEGs compared to controls, with 5,453 DEGs shared between both conditions (Figure 1B). Among the shared DEGs, genes such as *IGF1*, *SELENOP* and *PDK4* were significantly downregulated in both treatment groups relative to controls, while among others *CXCL1*, *CXCL3*, and *PTGS2* were significantly upregulated (Figure 1C). As expected, interferon-responsive genes such as *APOL1*, *APOL3*, *IRF1*, and *GBP2* were significantly upregulated in LPS/IFNγ conditions, while remaining unchanged in the LPS group (Figure 1C).

To further assess how LPS and LPS/IFNγ treatments relate to the DAM phenotype, we used a recently published DAM-gene list (Mancuso et al., 2024). Within our dataset 400 DAM-genes could be identified, out of which a total of 154 genes reached statistical significance when compared to the control group. Of these 154 DEGs, 77 DEGs were shared between both treatment groups, compared to the control group (Figure 1D). When compared to the control group, the two treatment groups shared both upregulated (e.g., *S100A9*, *NAMPT*, *ACSL1*) and downregulated (e.g., *KCNJ5*, *LRRC39*, *DHRS9*) DEGs. Interestingly, *TREM2*, *FABP3*, and *LPL* were downregulated in both treatment groups, with a more pronounced reduction by LPS/IFNγ than by LPS alone, suggesting an additive or synergistic effect of IFNγ (Figure 1E). Moreover, antigen-presenting genes (*HLA* gene family) were downregulated in the LPS group, while appearing largely unaffected, or even slightly increased following LPS/IFNγ treatment (Figure 1E). The expression pattern of *HLA* genes becomes even more obvious when comparing DAM DEGs in the LPS/IFNγ and LPS groups directly (Figure 1F). Furthermore, the LPS versus LPS/IFNγ comparison revealed differential expression of *TXN*, *PPA1*, *SCIN*, *CD68*, and *TNFAIP2*, which were significantly upregulated in LPS/IFNγ compared to LPS alone. In contrast, *SLC11A1*, *CD163*, and *CD9* were downregulated in LPS/IFNγ compared to LPS alone (Figure 1F). Overall, our data uncovers overlapping as well as distinct transcriptional signatures of LPS and LPS/IFNγ stimulation in microglia, underscoring the need for careful characterization of the effects of IFNγ and LPS when modeling inflammation.

### Temporal cytokine and chemokine expression dynamics are differentially affected by LPS, IFNγ, and their combination

**Figure 2:**
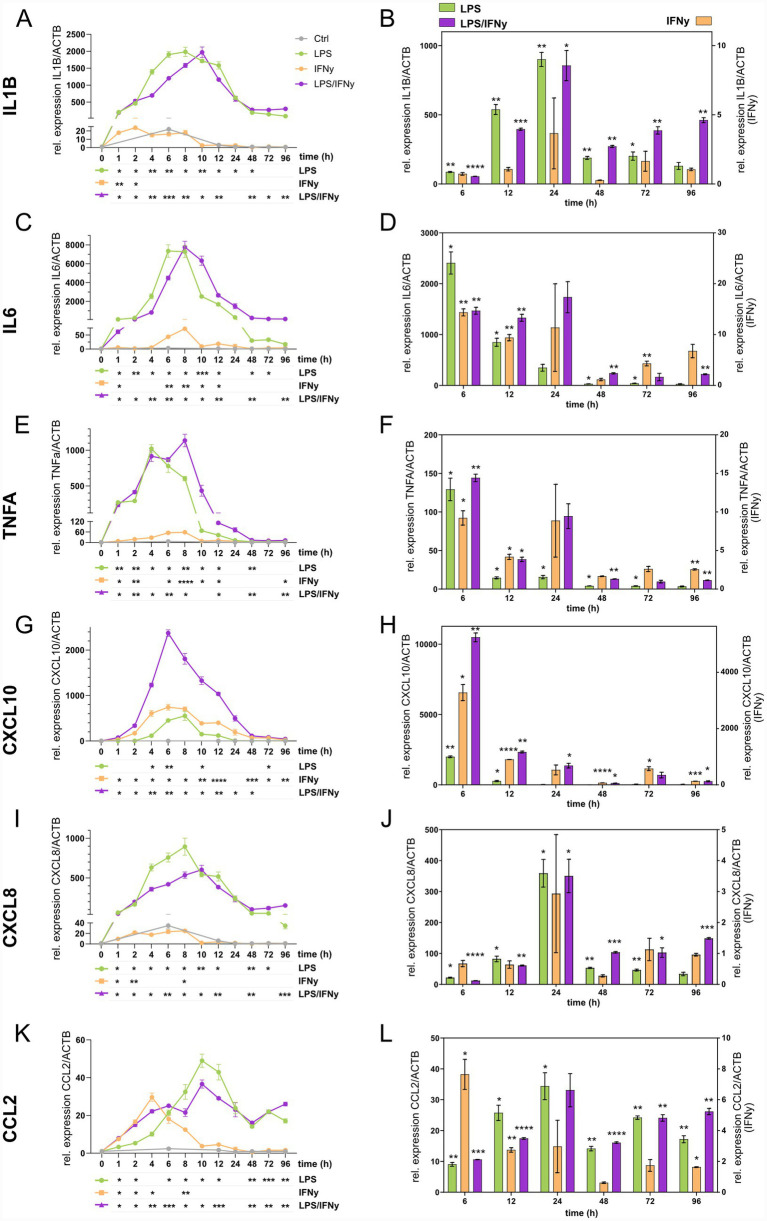
Temporal cytokine and chemokine gene expression dynamics in LPS, IFNγ, and LPS/IFNγ-treated microglia. Gene expression levels are presented as fold-change relative to timepoint 0 within each treatment group in panels **A,C,E,G,I,K** with statistical analyses for temporal changes displayed below each graph. Gene expression level comparisons at individual timepoints (6 h, 12 h, 24 h, 48 h, 72 h, 96 h) against CTRLs are shown in panels **B,D,F,H,J,L**, with LPS and LPS/IFNγ plotted on the left *y*-axis and IFNγ on the right *y*-axis. Genes analyzed are **(A,B)** *IL1B*, **(C,D)** *IL6*, **(E,F)** *TNFA*, **(G,H)** *CXCL10*, **(I,J)** *CXCL8*, **(K,L)** *CCL2*. *n* = 3 biological replicates of the same differentiation, mean ± SEM; two-way ANOVA with Dunnett’s multiple comparisons test, *p < 0.05, **p < 0.01, ***p < 0.001, ****p < 0.0001.

To investigate the temporal regulation of microglial inflammatory activation, we quantified the mRNA expression of key pro-inflammatory cytokines *IL1B* (Figures 2A,B), *IL6* (Figures 2C,D), and *TNFA* (Figures 2E,F), as well as chemokines *CXCL10* (Figures 2G,H), *CXCL8* (Figures 2I,J), and *CCL2* (Figures 2K,L). hiMG were stimulated with LPS, IFNγ, or their combination over a 96 h time course. Overall, all six genes were upregulated by the treatments, with variations in both timing and magnitude (Figures 2A,C,E,G,I,K). LPS and LPS/IFNγ induced strong mid-phase expression peaks (6–10 h) for *IL1B*, *IL6*, *TNFA*, *CXCL10*, and *CXCL8*, while *CCL2* expression peaked slightly later (10–12 h). IFNγ alone triggered earlier and more transient expression peaks (2–4 h) for *IL1B*, *CXCL8*, and *CCL2*, and intermediate expression peaks (6–8 h) for *IL6* and *CXCL10*. To further evaluate treatment effects, we compared the treatment groups to untreated controls at 6 h, 12 h, 24 h, 48 h, 72 h, and 96 h (Figures 2B,D,F,H,J,L). LPS and LPS/IFNγ (plotted on left *y*-axis) produced markedly stronger gene expression changes compared to controls than IFNγ alone did (plotted on right *y*-axis) with significant upregulation of all six genes at 6 h and 12 h. At later timepoints, expression levels declined, yet LPS/IFNγ maintained significant upregulation for most genes up to 96 h, whereas LPS alone only retained significance for *CCL2* at 96 h (Figure 2K). Collectively, these results indicate a long-lasting effect of the combination of LPS and IFNγ compared to LPS or IFNγ alone on microglial activation.

### Stimulation with LPS/IFNγ causes profound changes in microglial morphology

**Figure 3:**
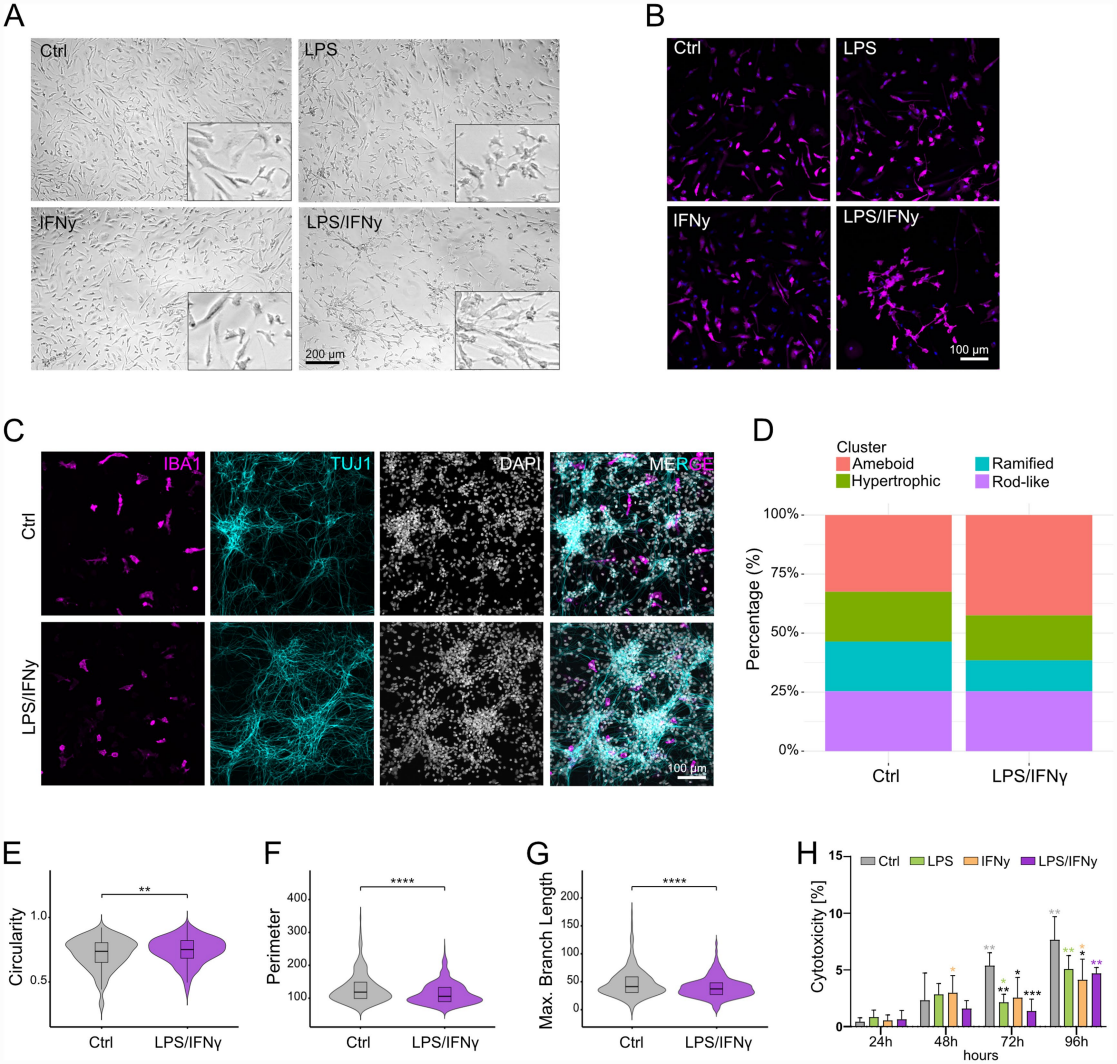
Analysis of microglial morphology changes in response to LPS, IFNγ, and LPS/IFNγ treatment. **(A)** Representative light microscopy pictures of Ctrl hiMG and hiMG stimulated with LPS, IFNγ, and LPS/IFNγ for 24 h. **(B)** Representative immunofluorescence pictures of IBA1-positive Ctrl hiMG and hiMG stimulated with LPS, IFNγ, and LPS/IFNγ for 24 h. **(C–G)** hiMG morphology analysis: **(C)** Representative immunofluorescence pictures of co-cultures of hiMG (IBA1) and hiPSC-derived neurons (TUJ1) with cell nuclei (DAPI), and **(D)** percentage of ameboid, hypertrophic, ramified, and rod-like microglia in Ctrl and LPS/IFNγ-stimulated co-cultures. Comparison between Ctrl and LPS/IFNγ-stimulated hiMG for **(E)** cell circularity, **(F)** cell perimeter, **(G)** maximum branch length. **(H)** Cytotoxicity assay performed for Ctrl and LPS-, IFNγ-, or LPS/IFNγ-stimulated hiMG at 24 h, 48 h, 72 h, and 96 h; black stars indicate significance within timepoints compared to Ctrl; colored stars indicate significance within groups compared to 24 h; *n* = 2 independent differentiations with biological triplicates; mean ± SD. For comparisons between two groups (panel **E–G**): *t*-test. For more than two groups (panel **H**): Two-way ANOVA with Tukey’s multiple comparisons test. *p < 0.05, **p < 0.01, ***p < 0.001, ****p < 0.0001.

We next investigated morphological features of microglial activation. While we could observe a shift toward the classical activated, ameboid, cellular morphology with LPS treatment, we did not observe major morphological changes with IFNγ treatment (Figures 3A,B; Supplementary Figure 2A). In contrast, distinct morphological phenotypes were observed with LPS/IFNγ treatment (Figures 3A,B), with microglial clustering observed as early as at 12 h post treatment (Supplementary Figure 2A). A similar clustering effect was observed in hiMG derived from a different hiPSC line (Supplementary Figure 2B). Due to the clustering, we could not assess microglial morphology in monocultures using our morphology analysis pipeline which is based on single cell analysis. To determine whether the observed clustering was specific to hiMG monocultures, we employed a co-culture system of hiMG and hiPSC-derived neurons. In addition, microglial morphological states are environment dependent and have been shown to be more complex in co-cultures with hiPSC-derived neurons. We analyzed microglial morphology in co-cultures using a recently published high-throughput analysis pipeline (Kim et al., 2024). hiPSC-derived neurons in co-cultures form mature networks and abundantly express synaptic vesicle glycoprotein 2A (SV2A) (Supplementary Figure 2C). Analysis of LPS/IFNγ-treated hiMG identified a shift in cell morphology with a greater percentage of ameboid and lower percentage of ramified cells compared to controls (Figures 3C,D). More specifically, microglia in LPS/IFNγ-treated co-cultures showed significantly increased circularity, while perimeter and maximum branch length were significantly decreased in comparison to microglia in control co-cultures (Figures 3E–G). Interestingly, we did not observe microglial clustering in the co-cultures (Figure 3C). To assess potential cytotoxic effects of the treatments on hiMG, we performed a cytotoxicity assay. As expected, a progressive increase in cytotoxicity can be observed for all groups over time (Figure 3H). However, all three treatment groups exhibited significantly lower cytotoxicity at 72 h and 96 h compared to controls at respective timepoints (Figure 3F). Altogether, our results underscore that microglial activation manifests through diverse morphological changes that depend heavily on environmental context.

### LPS-dependent increases and IFNγ-dependent decreases of microglia-secreted proteins

**Figure 4:**
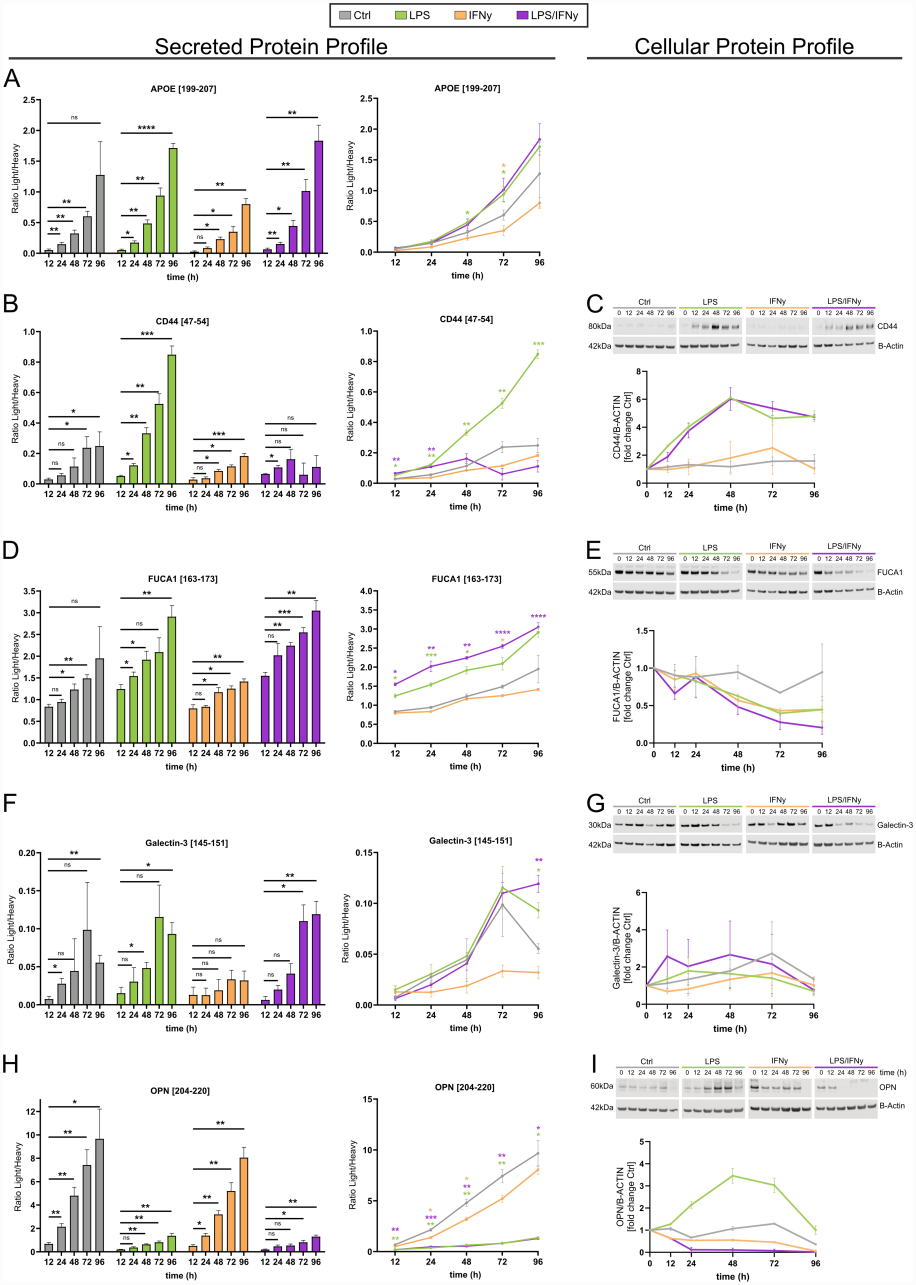
Temporal changes in the secreted and cellular protein profiles are affected by stimulation with LPS, IFNγ, and a combination of both. The temporal and treatment-dependent changes of ApoE **(A)**, CD44 **(B,C)**, FUCA1 **(D,E)**, Galectin-3 **(F,G)**, and OPN **(H,I)**, in the hiMG secreted protein profile **(A,B,D,F,H)** and cellular protein profile **(C,E,G,I)**. **(A,B,D,F,H)** Present the secreted protein profile as a comparison within each group to the 12 h timepoint (left) and as a comparison against Ctrl for each timepoint (right). **(C,E,G,I)** Present the cellular protein profile assessed by western blot and its analysis relative to B-Actin levels. Each treatment group is represented by color: Ctrl (gray), LPS (green), IFNγ (yellow), and LPS/IFNγ (purple). For the analysis of secreted proteins, *n* = 4 biological replicates of the same differentiation. Black stars indicate significance within treatment groups; colored stars indicate significance within timepoints between treatment groups; mean ± SD; two-way ANOVA with Tukey’s multiple comparisons test. For the analysis of cellular proteins, *n* = 2 independent differentiations; this sample size did not allow for meaningful statistical testing; mean ± SEM; *p < 0.05, **p < 0.01, ***p < 0.001, ****p < 0.0001.

For further characterization of the microglial activation profile, targeted mass spectrometry was used to measure a combination of microglial biomarker candidates in hiMG media. Levels of secreted proteins were compared with cellular protein levels measured by western blot. In total, the secreted and cellular levels of five proteins [apolipoprotein E (ApoE), phagocytic-glycoprotein 1 (CD44), alpha-L-fucosidase (FUCA1), Galectin-3, and osteopontin (OPN)] were evaluated (Figure 4). We further analyzed gene expression levels of the genes encoding for those proteins in our RNA-seq dataset (Supplementary Figure 3A). These proteins were chosen based on their relevance to microglial activation (De Schepper et al., 2023; Garcia-Revilla et al., 2022; Krasemann et al., 2017; Pesamaa et al., 2023; Supplementary Table 1).

Secreted levels of ApoE increased in a time-dependent manner, as observed for all conditions, including controls. Throughout all timepoints, secreted ApoE levels remained lowest in the IFNγ-treated group (Figure 4A). At 48 h and 72 h post LPS treatment, extracellular levels of ApoE were significantly higher than in the time-matched control group (Figure 4A). We could not compare these results to cellular ApoE levels since they could not be reliably detected by western blot. The soluble form of CD44 showed a slow increase in the media of controls and IFNγ-treated hiMG (Figure 4B). These modest changes were completely absent in the cell lysates, where the CD44 protein expression remained low throughout all timepoints in controls and IFNγ-treated hiMG (Figure 4C). The increase in secreted CD44 was most prominent in LPS-treated hiMG (Figure 4B). Furthermore, while the intracellular levels of CD44 correlated well with the extracellular CD44 levels in control, LPS, and the IFNγ group, there was a shift in these dynamics in the LPS/IFNγ group (Figures 4B,C). *CD44* gene expression was increased in LPS- and LPS/IFNγ-stimulated microglia (Supplementary Figure 3A). FUCA1 secretion increased in hiMG in a time-dependent manner, most prominently in LPS- and LPS/IFNγ-treated hiMG (Figure 4D). Interestingly, the intracellular levels of FUCA1 decreased over time with lowest levels at 96 h in the LPS- and LPS/IFNγ- treated hiMG (Figure 4E). Similarly, *FUCA1* gene expression was decreased in LPS- and LPS/IFNγ-stimulated microglia (Supplementary Figure 3A). Extracellular levels of Galectin-3 were low in general, as indicated by the ratio between light (endogenous) and heavy peptide (Figure 4F). However, an increasing trend was observed over time, which became significant for LPS- and LPS/IFNγ-treated hiMG, except for IFNγ-treated hiMG in which the Galectin-3 levels were lower than in controls (Figure 4F). The intracellular levels of Galectin-3 appeared variable with no significant changes (Figure 4G). Secreted levels of OPN were significantly decreased in the LPS and LPS/IFNγ groups compared to controls (Figure 4H). Controls and IFNγ-treated hiMG had approximately 8 times higher OPN levels at 96 h compared to 12 h (Figure 4H). Cellular levels of OPN as well as *SPP1* gene expression increased only in the LPS group (Figure 4I; Supplementary Figure 3A) while all other groups showed a decreasing trend over time.

**Figure 5:**
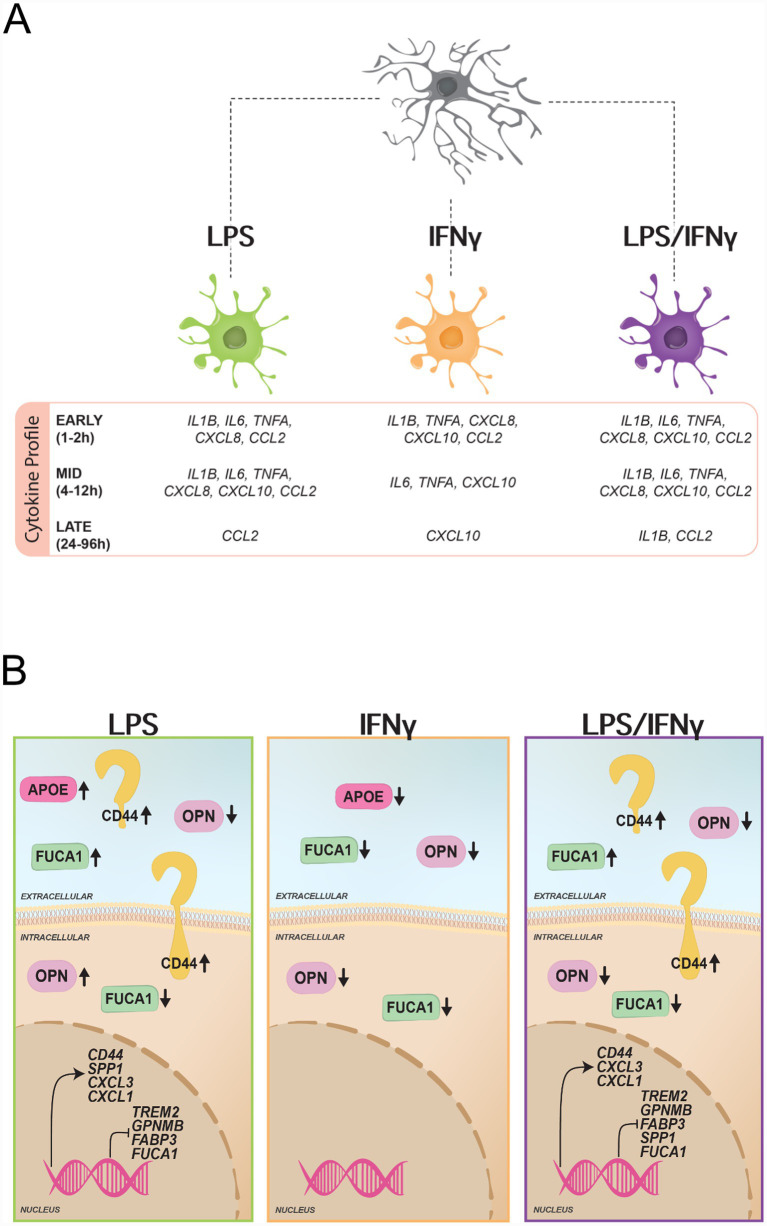
Summary of key changes in microglia upon LPS, IFNγ, and LPS/IFNγ treatment. **(A)** Illustration of key cytokine differences (>50% of timepoints reached significance) between the treatment groups divided into early (1–2 h), mid (4–12 h), and late (24–96 h) changes as identified by qPCR. **(B)** Illustration of key significant intracellular and extracellular protein as well as transcriptomic changes of the five microglia-relevant proteins measured as well as other microglia-relevant transcripts based on our RNA seq dataset (LPS and LPS/IFNγ).

In summary, our observations reveal distinct microglial protein expression-secretion dynamics as shown by: (1) the divergent intracellular and extracellular trajectories of FUCA1, where extracellular levels increase in a time-dependent manner, while intracellular protein levels concurrently decline (Figures 4D,E); (2) LPS-driven secretion, exemplified by ApoE, FUCA1, and Galectin-3 (Figures 4A,D,E); and (3) non-linear intracellular protein expression as demonstrated by the plateauing of CD44 and the transient peaking of OPN protein levels (Figures 4C,I). Key changes of microglia-relevant proteins and genes across treatment conditions are highlighted in Figure 5.

## Discussion

Microglia research has led to the identification of numerous activation signatures, forcing the field to shift from a binary characterization (resting versus activated and M1 versus M2) to a more comprehensive characterization of these cells (Paolicelli et al., 2022). Considering this expanded and complex view of microglial activation signatures, temporal and environmental conditions should also be considered when phenotyping microglia. Although LPS and IFNγ are widely used to induce microglial activation in experimental models (Stoberl et al., 2023), their shared and stimulus-specific effects, particularly in a temporal context, have not been systematically studied. Furthermore, the importance of understanding activation patterns and phenotypic states of human microglia is underscored by significant differences between microglia of different species (Geirsdottir et al., 2020; Mancuso et al., 2024). Our results address these knowledge gaps by demonstrating time- and treatment-dependent shifts in human microglial signatures.

Our transcriptomic data reveal clear differences between the microglial signatures following LPS and LPS/IFNγ treatment. Many of the top DEGs followed the same directionality across the two treatments, e.g., *IGF1*, *SELENOP*, and *PDK2* which were all strongly reduced in both treatment conditions, while *PTGS2*, *CXCL1*, and *CXCL3* were commonly upregulated. The LPS-induced reduction of microglial *IGF1* expression is likely to contribute to the toxic inflammatory phenotype that has been described in LPS *in-vivo* models (Jung et al., 2023; Zakaria et al., 2017; Zhao et al., 2019). The common upregulation of *PTGS2* (encoding prostaglandin G/H synthase 2), also known as *COX2*, might constitute a key driver of proinflammatory responses associated with detrimental effects (Li et al., 2025; Vijitruth et al., 2006). The increased expression of *PTGS2* is likely mediated by Tnf-α, induced by LPS-TLR4 interaction (Beutler, 2000; Kanda et al., 2017; Zakaria et al., 2017). When compared with our pre-selected reference set of human DAM genes (Mancuso et al., 2024), 154 DEGs were identified in either the LPS or the LPS/IFNγ group. Of these DAM DEGs, 50% were shared between both treatment conditions, while 34.4% of the DEGs were unique to the LPS/IFNγ-treated microglia (Figure 1D). This underscores the robust activation of microglia induced by both models, while the LPS/IFNγ-unique DEGs highlight the specific contribution of IFNγ in this model. Notably, typical DAM-markers such as *TREM2*, *GPNMB*, *FABP3*, and *LPL* were significantly downregulated in both treatment groups relative to the untreated control (Figure 1E). This observation is surprising, considering the frequently reported upregulation of these markers in different contexts of microglial activation (Keren-Shaul et al., 2017; Krasemann et al., 2017; Mancuso et al., 2024; Pesamaa et al., 2023). However, isolated pro-inflammatory stimulation of microglia using LPS has previously been shown to suppress TREM2 expression (Liu et al., 2020; Zhou et al., 2019). These findings further emphasize the complexity of microglial activation and inflammatory balance and suggest that a single experimental condition examined at a single timepoint is insufficient for a conclusive characterization of microglial states.

The temporal effects are demonstrated by the cytokine expression profiles, as assessed by qPCR for each of the three treatment groups compared to controls across several timepoints. The treatment- and time-dependent expression dynamics of *IL1B*, *IL6*, *TNFA*, and *CXCL8* were very similar, as they all peaked at the mid timepoint (4–12 h) in the LPS and LPS/IFNγ groups. This aligns with findings reported by others and suggests that these four factors, likely in coordination with NF-kB, act within pro-inflammatory pathways that together constitute a complex network of inflammatory mediators (Cambier et al., 2023; Ishijima and Nakajima, 2021). Although peaking at the mid timepoint, *IL1B* and *CXCL8* expression remained significantly higher throughout the late timepoints (24–96 h), while *TNFA* expression dropped markedly after the 12-h timepoint. In the LPS/IFNγ group, *IL6* expression was also clearly reduced at the late timepoints. However, in IFNγ-treated hiMG, *IL6* expression did not drop but showed a similar pattern to that of *IL1B* and *CXCL8*. This IFNγ-associated delayed phase supports the idea of IFNγ acting as a priming factor for sustained microglial activation (Hemmerich et al., 2022; Kann et al., 2022; Perry and Holmes, 2014).

Secretion of ApoE differed significantly across the different treatment conditions. LPS- and LPS/IFNγ-treated microglia released more ApoE than controls, while IFNγ-treated microglia released less. This finding is in line with previously reported results obtained from human monocytes, in which ApoE levels are post translationally regulated by IFNγ affecting both intracellular and extracellular ApoE levels following IFNγ treatment (Brand et al., 1993). CD44 is a transmembrane glycoprotein and cell surface receptor that binds hyaluronic acid, a receptor-ligand interaction that is promoted by LPS, while inhibited by IFNγ (Gee et al., 2003; Knupfer et al., 1997; Kryworuchko et al., 1999). Interestingly, *CD44* gene expression was increased at 24 h following LPS and LPS/IFNγ treatment which correlated with intracellular CD44 protein levels potentially suggesting a transcriptional regulation. Notably, CD44 acts as a receptor for OPN (Weber et al., 1996) and this interaction has been suggested to play a significant role in autoimmune diseases (Abel et al., 2006; Kim et al., 2004). Moreover, OPN is upregulated in several neuroinflammatory and neurodegenerative disorders (Rosmus et al., 2022) but the exact role in microglia in this context remains unclear. Binding of soluble OPN to CD44 promotes cell migration in macrophages (Lund et al., 2009; Marcondes et al., 2008). In microglia, OPN has been suggested to promote protective as well as detrimental responses suggesting that its function may depend on timing and context (Lawrence et al., 2024; Rabenstein et al., 2016; Rosmus et al., 2022). While OPN secretion was blunted in an LPS-dependent manner, intracellular OPN levels as well as *SPP1* gene expression increased with LPS and decreased with LPS/IFNγ treatment suggesting intracellular retention of OPN upon LPS treatment. Interestingly, intracellular FUCA1 levels as well as *FUCA1* gene expression decreased following LPS and LPS/IFNγ treatment while FUCA1 secretion increased. However, there seems to be a delay in the cellular FUCA1 decrease compared to gene expression suggesting that protein stability may play a role. Notably, for all targeted proteins, secreted levels were highest in the LPS and LPS/IFNγ groups, and lowest in the IFNγ group, except for extracellular OPN levels which were highest in the IFNγ group.

One limitation of this study is that beyond the 48-h time point, essential microglial factors in the media might become limiting. Even though we did not observe significant amounts of cell death or cytotoxicity, it could still alter microglial responses. Unexpectedly, we observed slightly lower cytotoxicity at later time points in all treatment groups compared to controls. Activated microglia are characterized by a unique metabolic state and have been suggested to be less dependent on CSF1R signaling (Krasemann et al., 2017) and IL-34 shortage. A key limitation of this study is the use of a single iPSC line for most experiments. Validation across additional iPSC lines is needed to confirm the generalizability of our results.

LPS/IFNγ-treated hiMG displayed profound morphological changes compared to controls. In monocultures, microglia clustered into networks whereas in co-cultures with hiPSC-derived neurons, microglia were less ramified and more amoeboid. LPS/IFNγ treatment did not influence the percentage of cells with rod-like morphology. The functional implications of rod-like microglia remain largely unclear (Holloway et al., 2019; Matsuba et al., 2025). Interestingly, a recent report linked rod-like morphology to microglial responses to type I interferons (Coburn et al., 2025). Clustering and filopodia formation of microglia has previously been observed upon combined IFNγ/TNFa treatment of primary rat microglia (Lively and Schlichter, 2018), in co-cultures of hiPSC-derived motor neurons and microglia upon LPS/IFNγ treatment (Vahsen et al., 2022), and in BV-2 mouse microglia upon LPS/IFNγ treatment (Sheng et al., 2011). Interestingly, clustering has also been observed in co-cultures with LPS treatment only (Haenseler et al., 2017). Clustering may be a way to facilitate intercellular communication and exchange between microglia, as was for example described for tunneling nanotubes (Scheiblich et al., 2021). Taken together, these observations should prompt further investigations into the link between microglial morphology and function and highlight that morphological changes heavily depend on environmental context.

Collectively, our results show that LPS and IFNγ induce distinct microglial activation profiles, each characterized by specific molecular signatures and temporal trajectories, rather than representing interchangeable pro-inflammatory stimuli. These findings emphasize that both the choice of stimulus and the duration of exposure critically shape microglial phenotype, with important implications for experimental design and for the interpretation of microglial behavior in disease models. Recognizing these stimulus-specific and time-dependent factors will be essential for refining *in vitro* paradigms and for developing more accurate models of neuroinflammation.

## Data Availability

The datasets presented in this study can be found in online repositories. The names of the repository/repositories and accession number(s) can be found in the article/Supplementary material.

## Supplementary material

SUPPLEMENTARY FIGURE 1 **(A)** Sample distance heatmap from RNAseq analysis, *n* = 2–3 biological replicates of the same differentiation.

SUPPLEMENTARY FIGURE 2 **(A)** Representative light microscopy pictures of Ctrl hiMG and hiMG stimulated with LPS, IFNg, or LPS/IFNg for 12 h, 24 h, 48 h, 72 h, and 96 h. **(B)** Representative light microscopy pictures of hiMG derived from the hiPS cell line BIONC, stimulated with LPS, IFNg, or LPS/IFNg for 24 h. **(C)** Representative immunofluorescence pictures of co-cultures of hiMG (IBA1) and hiPSC-derived neurons with synaptic vesicles (SV2A) with cell nuclei (DAPI). **(D)** Heatmap of features defining clusters 1–4 in morphology analysis. **(E)** Heatmap for correlation between PCs and features in morphology analysis. Spearman rank order correlation with no multiple testing correction. **p* < 0.05.

SUPPLEMENTARY FIGURE 3 **(A)** Heatmap showing rlog-transformed RNA-seq expression of selected genes, with expression values centered to the mean across all samples (per row); *n* = 2–3 biological replicates of the same differentiation.

SUPPLEMENTARY TABLE 1 Microglia-relevant proteins measured with targeted mass spectrometry. Overview of targeted proteins, their respective accession number and gene name, peptide position within the full amino acid sequence, and their relevance to microglia with respective references. In total, seven peptides, representing five proteins, were evaluated.
