# Supplementary figures and images for: The temporal and stimuli-specific effects of LPS and IFNγ on microglial activation
Source: Front Aging Neurosci. 2026 Jan 29;18:1756410. doi: 10.3389/fnagi.2026.1756410 (PMC12894321; doi:10.3389/fnagi.2026.1756410)

A

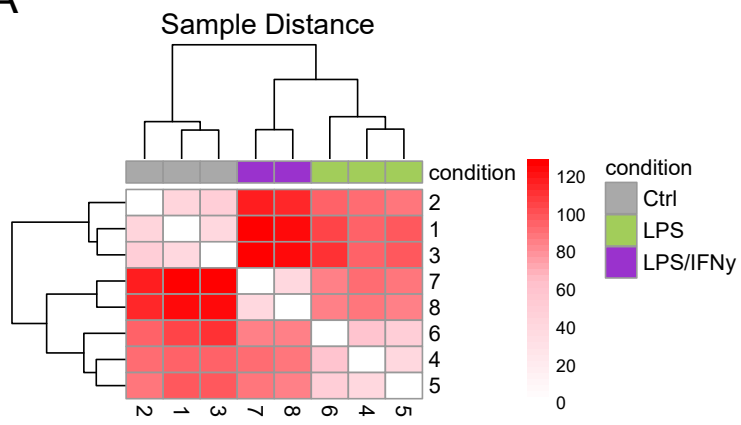

Supplement: Supplementary file 1 [file Data_Sheet_1.pdf]

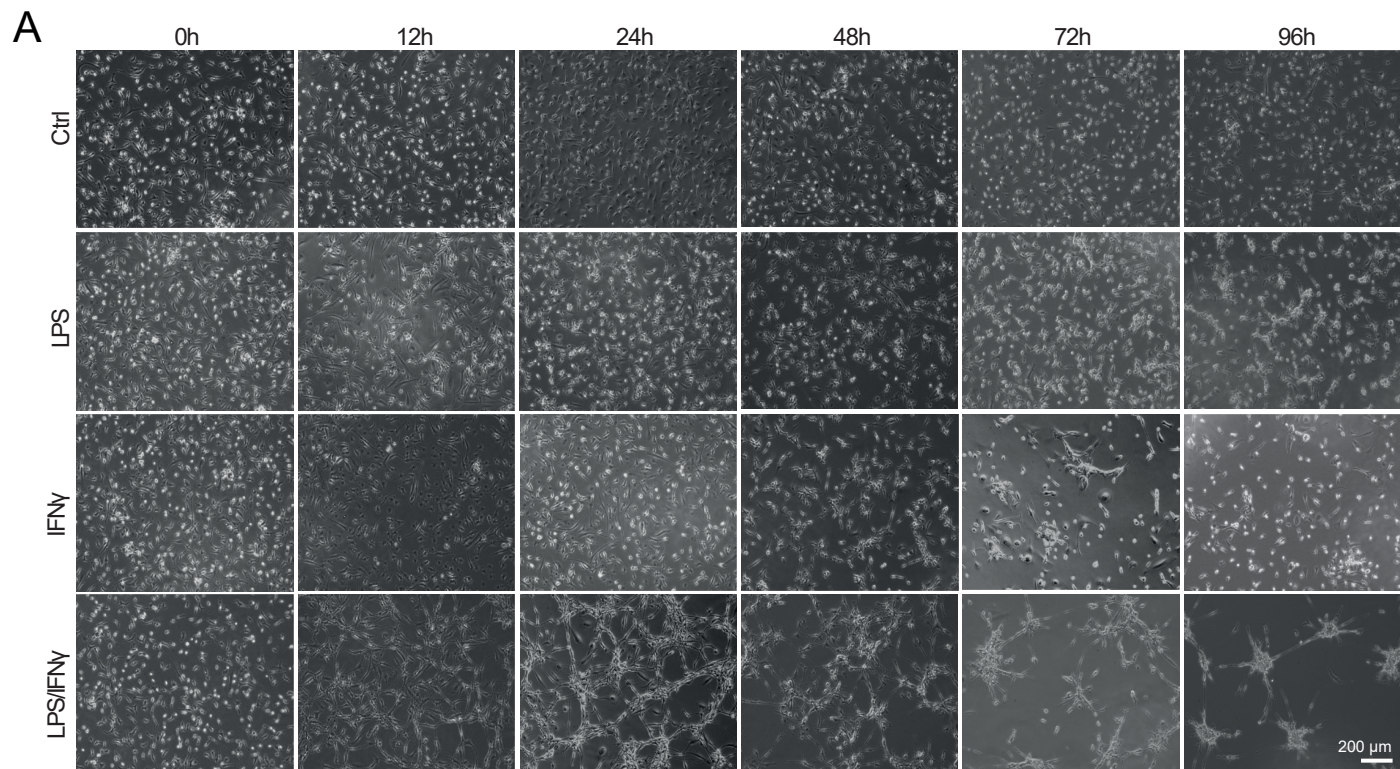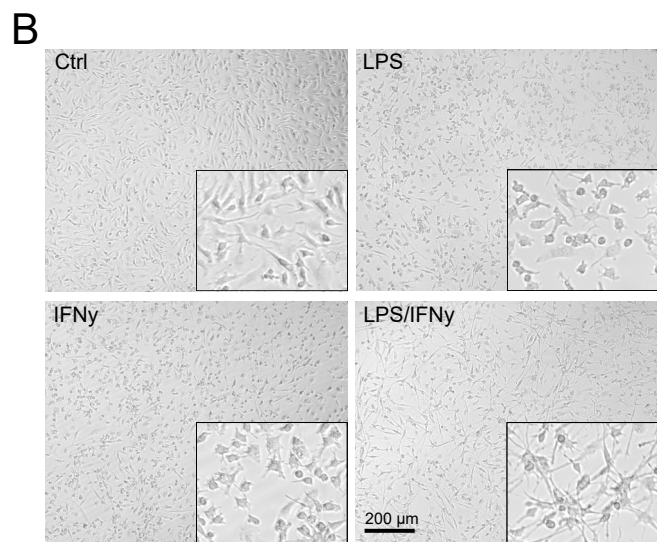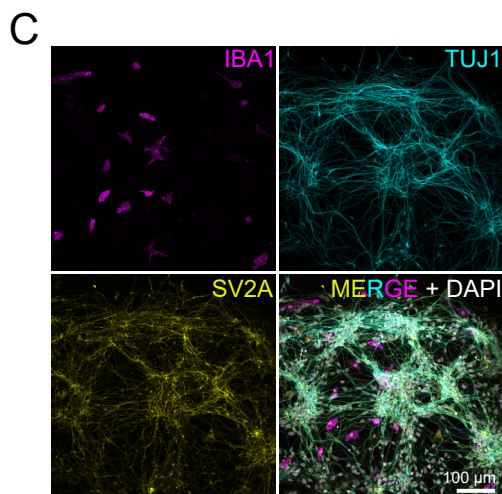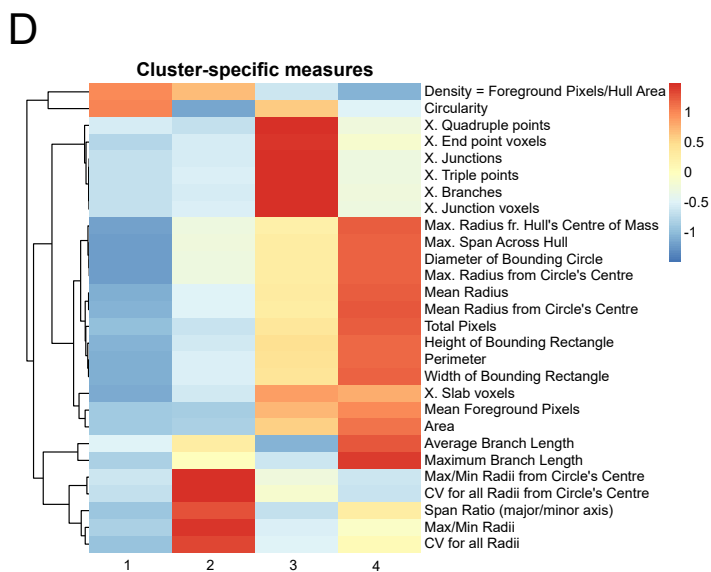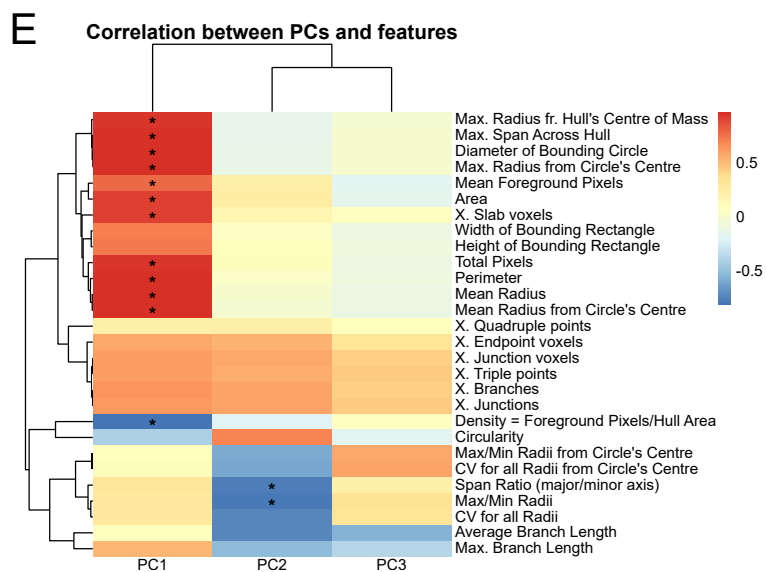

Supplement: Supplementary file 3 [file Data_Sheet_3.pdf]

A

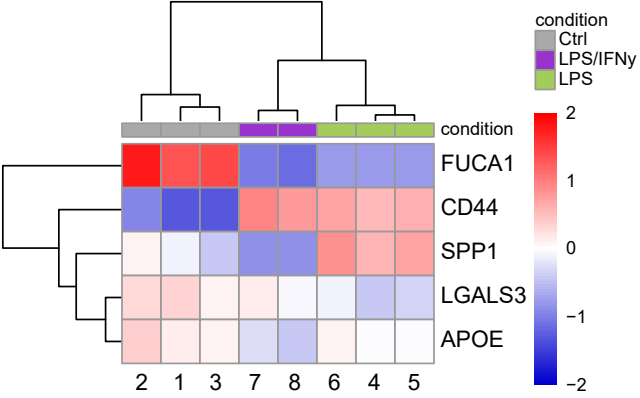

Supplement: Supplementary file 4 [file Data_Sheet_4.pdf]
