# Supplementary material for: The temporal and stimuli-specific effects of LPS and IFNγ on microglial activation
Source: Front Aging Neurosci. 2026 Jan 29;18:1756410. doi: 10.3389/fnagi.2026.1756410 (PMC12894321; doi:10.3389/fnagi.2026.1756410)
Supplement: Supplementary file 2 [file Data_Sheet_2.pdf]

PROTEIN	ACCESSION NUMBER	GENE NAME	PEPTIDE POSITION	RELEVANCE TO MICROGLIA	REFERENCES
Phagocytic-glycoprotein 1 (CD44)	P16070	*CD44*	47-54	Microglial activation	Pesämaa et al., 2023
Apolipoprotein E (ApoE)	P02649	*APOE*	199-207	Microglial activation	Krasemann et al., 2017
Alpha-L-fucosidase (FUCA1)	P04066	*FUCA1*	163-173; 344-354	Microglial activation	Pesämaa et al., 2023
Osteopontin (OPN)	P10451	*SPP1*	204-220; 302-314	Microglial activation	De Schepper et al., 2023
Galectin-3	P17931	*LGALS3*	145-151	Microglial activation	García-Revilla et al., 2022
